# Burn injuries related to E-cigarettes reported to poison control centers in the United States, 2010–2019

**DOI:** 10.1186/s40621-020-00263-0

**Published:** 2020-07-20

**Authors:** Baoguang Wang, Sherry T. Liu, Brian Rostron, Camille Hayslett

**Affiliations:** United States Food and Drug Administration, Center for Tobacco Products, Office of Science, 10903 New Hampshire Avenue, Silver Spring, MD 20993 USA

**Keywords:** Electronic cigarette, E-cigarette, Burn, Injury

## Abstract

**Background:**

United States (U.S.) national data indicate that 2035 individuals with burn injuries from e-cigarette explosions presented to U.S. hospital emergency departments (EDs) in 2015–2017. This national estimate is valuable for understanding the burden of burn injuries from e-cigarette explosions among individuals who presented to EDs. However, little is known about individuals who experienced e-cigarette-related burns but may not present to EDs or health care facilities.

**Findings:**

We analyzed data from the National Poison Data System (NPDS) to describe frequency and characteristics of e-cigarette-related burn cases in the U.S. in 2010–2019. NPDS contains information collected during telephone calls to poison control centers (PCCs) across the U.S., including e-cigarette-related burns and other unintended events. During 2010–2019, 19,306 exposure cases involving e-cigarettes were documented in NPDS. Of those, 69 were burn cases. The number of burn cases increased from one in 2011 to a peak of 26 in 2016, then decreased to three in 2019. The majority of the burn cases occurred among young adults aged 18–24 years (29.0%; *n* = 20) and adults aged 25 years or older (43.5%; *n* = 30); 14.4% (*n* = 10) occurred among individuals ≤17 years old. Of the 69 burn cases, 5.8% (*n* = 4) were admitted to a hospital; 65.2% (*n* = 45) were treated and released; 15.9% (*n* = 11) were not referred to a health care facility (HCF); 4.4% (*n* = 3) refused referral or did not arrive at an HCF; and 8.7% (*n* = 6) were lost to follow-up or left the HCF against medical advice. Nearly one-third (30.4%; *n* = 21) of the cases had a minor effect (symptoms resolved quickly), 47.8% (*n* = 33) had a moderate effect (symptoms were more pronounced and prolonged than in minor cases, but not life-threatening), and 2.9% (*n* = 2) had a major effect (life-threatening symptoms).

**Conclusions:**

Approximately one-fifth of e-cigarette-related burn cases reported to PCCs were not referred to or did not arrive at an HCF. Some burn cases had serious medical outcomes. The burn cases mostly affected young adults and adults aged 25 years or older. The number of burn cases in NPDS represents a small portion of e-cigarette-related burn cases but it can serve as a complementary data source to traditional injury surveillance systems.

## Background

Despite the increasing popularity of e-cigarette products, particularly among youth and young adults (Cullen et al., 2019; Wang et al., 2018; Creamer et al., 2019; Corey et al., 2018), potential health risks have not been fully characterized. Acute adverse effects associated with e-cigarette use, ranging from burn and explosion injuries to seizures and lung injuries, have been reported (Corey et al., 2018; Rudy & Durmowicz, 2016; Brownson et al., 2016; Rossheim et al., 2019; Faulcon et al., 2020; Perrine et al., 2019). Rudy and Durmowicz (Rudy & Durmowicz, 2016) identified 92 e-cigarette-related overheating, fire, or explosion cases between 2009 and 2015 from various data sources, including 13 cases reported to the United States (U.S.) Department of Health and Human Services, Food and Drug Administration (FDA), Center for Tobacco Products (CTP) Safety Reporting Portal (FDA, 2019a) and 21 cases documented in the National Electronic Injury Surveillance System (NEISS). Brownson et al. (Brownson et al., 2016) reported 15 patients with explosion injuries from e-cigarettes treated in one medical center between 2015 and 2016. Of these 15 patients, 80% had flame burns, 33% had chemical burns, and 27% had blast injuries. Two research teams analyzed nationally representative data from NEISS and provided national estimates of e-cigarette-related burn and explosion injuries presenting to U.S. hospital emergency departments (EDs). They estimated that 1007 individuals with battery-related burn injuries presented to EDs in 2016 (Corey et al., 2018) and 2035 individuals with burns and injuries from e-cigarette explosion presented to EDs from 2015 to 2017 (Corey et al., 2018; Rossheim et al., 2019). Although these national estimates are valuable for understanding the burden of e-cigarette-related burn and explosion injuries, the information is limited to individuals who presented to EDs with such injuries. Little is known about individuals who experienced e-cigarette-relate burns but may not have presented to EDs or health care facilities.

Unlike NEISS, which collects data from EDs of approximately 100 U.S. hospitals selected as a probability sample of more than 5000 U.S. hospitals with at least five beds and an ED, the National Poison Data System (NPDS) gathers information reported to U.S. poison control centers (PCCs) regarding injuries and poisoning exposures in individuals who may or may not present to EDs or health care facilities. This study describes the frequency and characteristics of e-cigarette-related burn cases documented in NPDS from 2010 to 2019.

## Methods

### NPDS data

In 2019, we analyzed data from NPDS, a data repository of injury and poisoning exposure calls to PCCs in the U.S. Details on NPDS have been described elsewhere (Wang & Rostron, 2017). Briefly, NPDS stores information on injury and poisoning events involving more than 437,000 products, including e-cigarettes and other tobacco products, reported to PCCs (Gummin et al., 2018). Each product is assigned a product code and a generic code. Product codes for e-cigarette devices and liquids became available in 2010 (Gummin et al., 2017; Bronstein et al., 2011; Bronstein et al., 2012). During each telephone call to a PCC, the following information is collected and documented using a structured computer program: information on caller location, exposure site, demographic characteristics of person experiencing poisoning exposure or injury, products involved, clinical effect (i.e., symptoms), level of care at a health care facility (HCF), and medical outcome. The information collected during each telephone call is uploaded to NPDS automatically in approximately 8 min (Gummin et al., 2018).

PCCs are known for providing information on substance toxicity and advice on poisoning exposure management. However, they respond to telephone calls regarding burns as well. NPDS codes burns as clinical effects, the same way it codes other symptoms, such as vomiting, headache, or abdominal pain. There is a built-in drop-down list of 131 clinical effects in the NPDS database, including superficial burns, second- or third-degree burns, oral burns, and burns that were not specified. Therefore, data on burns are systematically collected across all PCCs. We identified e-cigarette-related cases by selecting all e-cigarette product codes in NPDS. We then identified e-cigarette-related burn cases by selecting all e-cigarette product codes and clinical effects of burns in NPDS. This study focused specifically on e-cigarette-related burns documented as clinical effects in NPDS: superficial burns, second- or third-degree burns, oral burns, and burns that were not specified.

We analyzed NPDS data on burn cases associated with e-cigarettes only (i.e., no other substances were involved) that were reported to PCCs between July 1, 2010 and June 30, 2019. The following cases were excluded from the analysis: confirmed non-exposure (*n* = 61) or burns unrelated to e-cigarettes (*n* = 717); and burns reported from a foreign country (*n* = 10) or overseas diplomatic personnel or US military (*n* = 4).

### Case narratives

In addition to NPDS data, we reviewed case narratives (i.e., free-text notes written by PCC staff for each telephone call to a PCC) to explore context and circumstances of the e-cigarette-related burn cases. The lead author (BW) and a co-author (CH) of this manuscript reviewed all case narratives of burn cases identified for this study and extracted information on pre-defined characteristics. These characteristics included: whether e-cigarette explosion occurred (yes or no), type of burns (i.e., thermal burn, chemical burn, or both), affected body part(s), and whether a leaking e-cigarette product (leaking e-liquid) was involved. Information on these characteristics was documented in the free-text case notes of the case narratives with great variation from PCC to PCC, particularly for type of burn. The information on type of burn could be documented in an initial note or follow-up notes and could be noted explicitly or inexplicitly. Inter-reviewer discrepancies on case narrative findings were reconciled by another co-author (STL).

We computed descriptive statistics (i.e., number and percentage of cases) on these topics and calculated Cohen’s Kappa statistics to assess inter-reviewer reliability. We conducted data analysis using SAS Version 9.3 (SAS Institute Inc., Cary, NC, USA).

## Results

### NPDS data

**Fig. 1:**
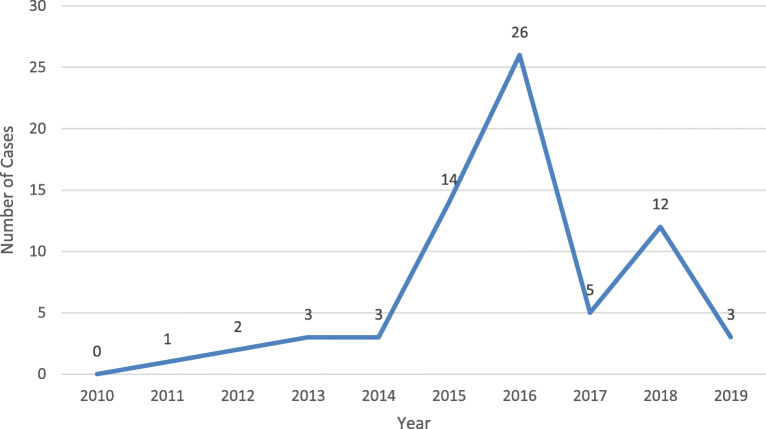
Number of e-cigarette-related burn cases reported to poison control centers in the United States, 2010–2019

During 2010–2019, 19,306 exposure cases involving e-cigarettes were documented in NPDS. Of the 19,306 cases, 69 were e-cigarette-related burn cases. The annual number of e-cigarette-related burn cases increased from one in 2011 to a peak of 26 in 2016, then decreased to three in 2019 (Fig. 1). Table 1 shows NPDS data on the characteristics of these cases. Overall, the majority of burn cases occurred among young adults aged 18–24 years (29.0%) and adults aged 25 years or older (43.5%). More than one-fifth of the cases occurred among children younger than age 5 years (2.9%), adolescents aged 12–17 years (11.5%), and individuals without exact age information (13.0%) (eight individuals were adults ≥20 years old and one individual had no information on age). There were more male cases (56.5%) than female cases. The majority (69.6%) of the burn cases were reported by health care professionals. Of the 69 burn cases, 5.8% (*n* = 4) were admitted to a hospital; 65.2% (*n* = 45) were treated, evaluated, and released; 15.9% (*n* = 11) were not referred to an HCF, but include two cases with a moderate effect that were managed on site at a non-HCF; and 4.4% (*n* = 3) refused referral or did not arrive at an HCF. More than half (58.0%; *n* = 40) of the burn cases had a superficial burn; 36.2% (*n* = 25) had a second- or third-degree burn. Nearly one-third (30.4%; *n* = 21) of the burn cases were minor (i.e., symptoms were minimally bothersome to the patient and they usually resolve rapidly), 47.8% (*n* = 33) were moderate (i.e., symptoms were more pronounced and prolonged than minor cases, but not life-threatening), and 2.9% (*n* = 2) were major (i.e., symptoms were life-threatening). Approximately two-thirds (61.9%; *n* = 13) of burn cases with a minor medical outcome and 81.8% (*n* = 27) of burn cases with a moderate medical outcome were treated, evaluated, and released (Table 2).

**Table 1:** Characteristics of e-cigarette-related burn cases reported to poison control centers in the United States, 2010–2019

Characteristics	Number (%) of E-Cigarette-Related Burn Cases
	Overall (*n* = 69)
Information from National Poison Data System	
Age (Years)	
< 5	2 (2.9%)
5–11	0 (0.0%)
12–17	8 (11.5%)
18–24	20 (29.0%)
25 +	30 (43.5%)
Unknown or Exact Age Unknown	9 (13.0%)
Gender	
Female	28 (40.6%)
Male	39 (56.5%)
Unknown	2 (2.9%)
Call Site	
Heath Care Facility (HCF)	48 (69.6%)
Own Residence	20 (29.0%)
Unknown	1 (1.5%)
Level of Care at HCF	
Admitted to Hospital	4 (5.8%)
Treated, Evaluated, and Released	45 (65.2%)
Refused Referral or Did Not Arrive at HCF	3 (4.4%)
Lost to Follow-Up or Left AMA	6 (8.7%)
Not Referred	11 (15.9%)
Clinical Effect	
Superficial Burn	40 (58.0%)
2nd- 3rd Degree Burns	25 (36.2%)
Oral Burns	5 (7.3%)
Burns, Not Specified	7 (10.1%)
Medical Outcome^a^	
No Effect	0 (0.0%)
Minor Effect	21 (30.4%)
Moderate Effect	33 (47.8%)
Major Effect	2 (2.9%)
Death	0 (0.0%)
Not Followed or Unable to Follow	13 (18.8%)
Information from Case Narrative Review	
Involved ENDS Explosion	
No	24 (34.8%)
Yes	45 (65.2%)
Involved Leaking	
Not Mentioned	63 (91.3%)
Yes	6 (8.7%)
Type of Burn	
Thermal	42 (60.9%)
Chemical	21 (30.4%)
Both Thermal and Chemical	5 (7.2%)
Not Specified	1 (1.4%)
Body Part Burned	
More than One Body Part	18 (26.1%)
Face Only (including eyes, nose, lip, and tongue)	23 (33.3%)
Leg/Thigh Only	13 (18.8%)
Hand Only	10 (14.5%)
Shoulder/Chest Only	1 (1.4%)
Genitals Only	1 (1.4%)
Not Specified	3 (4.3%)

^a^Medical outcome includes no effect (i.e., the patient did not develop any symptoms), minor effect (i.e., the patient exhibited some symptoms as a result of the exposure, but they were minimally bothersome to the patient and they usually resolve rapidly), moderate effect (i.e., the patient exhibited symptoms, which were more pronounced and prolonged than minor effect, but not life-threatening), and major effect (i.e., the patient developed symptoms which were life-threatening)

**Table 2:** Level of care at a health care facility by medical outcome for e-cigarette-related burn cases reported to poison control centers in the United States, 2010–2019

Level of Care at Health Care Facility (HCF)	Medical Outcome^b^					
	Total (*n* = 69)	No Effect (*n* = 0)	Minor Effect (*n* = 21)	Moderate Effect (*n* = 33)	Major Effect (*n* = 2)	Not Followed or Unable to Follow (*n* = 13)
Admitted to Hospital	4 (5.8%)	0 (0.0%)	0 (0.0%)	3 (9.1%)	0 (0.0%)	1 (7.7%)
Treated, Evaluated, and Released	45 (65.2%)	0 (0.0%)	13 (61.9%)	27 (81.8%)	1 (50.0%)	4 (30.8%)
Refused Referral or Did Not Arrive at HCF	3 (4.4%)	0 (0.0%)	1 (4.8%)	1 (3.0%)	0 (0.0%)	1 (7.7%)
Lost to Follow-Up or Left AMA^a^	6 (8.7%)	0 (0.0%)	1 (4.8%)	0 (0.0%)	1 (50.0%)	4 (30.8%)
Not Referred	11 (15.9%)	0 (0.0%)	6 (28.6%)	2 (6.1%)	0 (0.0%)	3 (23.1%)

^a^*AMA* Against medical advice

^b^Medical outcome includes no effect (i.e., the patient did not develop any symptoms), minor effect (i.e., the patient exhibited some symptoms as a result of the exposure, but they were minimally bothersome to the patient and they usually resolve rapidly), moderate effect (i.e., the patient exhibited symptoms, which were more pronounced and prolonged than minor effect, but not life-threatening), and major effect (i.e., the patient developed symptoms which were life-threatening)

### Case narratives

We reviewed case narratives for all 69 burn cases. The review of the case narratives revealed that approximately two-thirds (65.2%; *n* = 45) of the cases involved e-cigarette explosion (Table 1). Nearly two-thirds (60.9%; *n* = 42) of the cases had thermal burns; 30.4% (*n* = 21) of the cases had chemical burns; and 7.2% (*n* = 5) had both thermal and chemical burns. The most frequently reported body part burned was the face (33.3%; *n* = 23), including eyes, nose, lip, and tongue. About one-fourth (26.1%; *n* = 18) of the cases had burns on more than one body part, followed by burns to only leg/thigh (18.8%; *n* = 13), hands (14.5%; *n* = 10), shoulder/chest (1.4%; *n* = 1), and genitals (1.4%; *n* = 1). Of all the case narratives we reviewed, six indicated that a leaking product was involved. Agreement between the two reviewers was high for whether e-cigarette explosion occurred (Kappa value = 0.84), whether a leaking e-cigarette was involved (Kappa value = 0.84), and body part burned (Kappa values = 0.89). Kappa value for type of burn was 0.41.

## Discussion

Data from NPDS, a national surveillance system, indicate that approximately one-fifth of e-cigarette-related burn cases reported to PCCs were not referred to or did not arrive at HCFs. This information is complementary to the findings from previous studies on e-cigarette-related burn injury cases presented to EDs (Corey et al., 2018; Rossheim et al., 2019) and is helpful in understanding the overall burden of e-cigarette-related burn injuries.

Previous studies estimate that more than 1000 e-cigarette explosion and burn injuries occur in the U.S. per year (Corey et al., 2018; Rossheim et al., 2019). Only 69 e-cigarette-related burn cases were documented in NPDS from 2010 to 2019. This small number of burn cases in NPDS may reflect a substantial underreporting commonly suffered by surveillance systems relying on voluntary reporting. Since PCCs’ primary goal is to help individuals manage poisoning exposures, many burn cases that required immediate medical attention probably bypassed calling PCCs for help and presented to EDs directly.

We observed the largest number of e-cigarette-related burn cases in 2016 followed by a decline in 2017, a similar observation as noted by Rossheim et al. in their study on e-cigarette-related burn and explosion injuries presenting to EDs (Rossheim et al., 2019). The decline in the number of burn cases from 2016 to 2017 we observed was more pronounced than that reported by Rosseheim et al. This decline coincided with the following events occurring around the time: FDA provided an online education program entitled “Tips to Help Avoid “Vape” Battery Explosions” (FDA, 2017); the U.S. Fire Department published a report of e-cigarette-related fires and explosions in the U.S. and provided information on appropriate use of e-cigarettes (McKenna, 2017); and several case series reports of explosion injuries from e-cigarettes in the U.S. were published to alert the public about potential dangers of e-cigarette explosions, including two reports by FDA (Rudy & Durmowicz, 2016; Brownson et al., 2016; Durmowicz et al., 2016). As voluntary reports to PCCs involving e-cigarette-related burns can be affected by several factors, such as media coverage and awareness level of free services provided by PCCs, it is unclear whether media and published reports influenced individuals’ awareness of e-cigarette-related burns, leading to reports to PCCs. Specific reasons for the decline in the number of cases from 2016 to 2017 are difficult to identify with certainty. Given the small number of cases and limitations of NPDS as a passive surveillance system, caution is warranted when interpreting the findings of this study.

One of the unique features of PCCs is their case narratives documenting details on each case, including the circumstances of exposures, the development and progress of the case, treatment and management, and outcome. From the review of case narratives for all 69 burn cases, we noted that six mentioned leaking e-liquids. To our knowledge, this is the first study to suggest that leaky e-cigarettes may be involved in burn injuries. As the landscape of e-cigarettes is evolving rapidly, active surveillance of NPDS, NEISS, social media data, and other data is important for identifying health risks of these products and informing efforts to prevent harm associated with emerging tobacco products (Trigger & Coleman, 2019; Chang et al., 2019; Wang et al., 2019). FDA has taken steps to address some safety issues associated with e-cigarettes and e-liquids. In November 2019, FDA issued a guidance about its compliance policy for limited safety modifications to certain marketed tobacco products, including battery-operated tobacco products to address battery injury concerns in order to better protect consumers (FDA, 2019b).

One of the major limitations of this study is underreporting, as discussed previously. In addition, self-reported information on burn characteristics is subject to reporting bias, particularly for the burn cases reported by individuals from their own residences, which represent nearly one-third of the burn cases. However, a unique feature of NPDS is the use of follow-up contacts to verify and update information initially reported to PCCs to ensure the accuracy of the information in addition to monitoring case progress, collecting additional information, and determining the medical outcome of the cases (Gummin et al., 2018). Finally, lack of a standard format for case narratives may have resulted in somewhat inconsistent case narrative findings between the two reviewers, particularly for information on type of burn. Unlike the information on explosion and body part burned, type of burn was not directly stated in the case narratives and details sometimes were buried in the progress notes (i.e., follow-up notes) rather than in the initial note. The wide variation in case narratives could be the primary reason for the relatively low inter-reviewer reliability for some of the case narrative findings, such as type of burn. However, the third reviewer reviewed the case narratives to reconcile the discrepancies and provided final coding for the data analysis.

This study analyzed data from a national surveillance system to describe the frequency and characteristics of e-cigarette-related burn cases as well as the proportion of those cases that did not present to health care facilities. The number of burn cases documented in NPDS represents a small proportion of total e-cigarette-related burn cases, but it can serve as a complementary data source to other traditional injury surveillance systems, such as NEISS and FDA Safety Reporting Portal (www.safetyreporting.hhs.gov). Findings from case narratives provide additional contextual information that may inform tobacco product labeling, the development of tobacco product standards, and health communication and education programs aimed at preventing e-cigarette-related burns.

## Data Availability

Data for this study are available through contracts with the American Association of Poison Control Centers (www.aapcc.org).

## Abbreviations

CTP: Center for Tobacco Products
ED: Emergency Department
FDA: Food and Drug Administration
HCF: Health Care Facility
NEISS: National Electronic Injury Surveillance System
NPDS: National Poison Data System
PCC: Poison Control Center
U.S.: United States
